# Organization of the macroinvertebrate community in a tropical annual agroecosystem into modules

**DOI:** 10.1371/journal.pone.0289103

**Published:** 2023-08-03

**Authors:** David A. Andow, Eliana M. G. Fontes, Carmen S. S. Pires, Débora P. Paula

**Affiliations:** 1 Embrapa Recursos Genéticos e Biotecnologia, Brasília, Federal District, Brazil; 2 University of Minnesota, St. Paul, Minnesota, United States of America; Zhejiang University, CHINA

## Abstract

The structure of macroinvertebrate communities in agroecosystems has been assumed to be modular and organized around key herbivore pests. We characterized the macroinvertebrate community in the annual organic brassica agroecosystem in tropical central Brazil to determine if the community was a random assemblage of independent populations or was organized into repeatable multi-species components. We sampled 36 macroinvertebrate taxa associated with six organic brassica farms at biweekly intervals during the dry season during two years in the Distrito Federal, Brazil. We used an unconstrained ordination based on latent variable modeling (*boral*) with negative binomial population counts to analyze community composition independent of variation in sample abundance. We evaluated observed community structure by comparing it with randomized alternatives. We found that the community was not a random assemblage and consistently organized itself into two modules based around the major herbivores; one with lepidoptera and whiteflies and their associated natural enemies which was gradually replaced during the season by one with brassica aphids, aphid parasitoids and coccinellids. This analysis suggests that the historical and present-day focus on pest herbivores and their associated species in agroecosystems may be justified based on community structure.

## Introduction

Annual agroecosystems have been used to examine many ecological questions because they are amenable to experimentation and replication and have lower species richness than is typical of natural ecosystems. Although some intensive studies of annual agroecosystems have found >500 macroinvertebrate species [e.g., 1, 2], a lower species richness is more commonly found. If we consider that each species could have its own unique response to the abiotic and biotic environment a complete theoretical description of these communities would require one equation for each species. Thus, in annual agroecosystems, the macroinvertebrate community is a high-dimensional system. In practice, however, most studies on macroinvertebrates in annual agroecosystems have focused on one or a few key pest populations and their “life systems” with the goal of understanding pest biology and improving pest control. A life system is the common natural enemies and main abiotic factors associated with a pest or pest complex [3]. Although this approach has resulted in considerable pest control successes, it begs the question if such a focus can be justified from the characteristics of the structure of the broader macroinvertebrate community in the agroecosystem.

Recent work in community ecology has focused on describing community organization and evaluating its consequences. Theoretical and empirical work has suggested that communities are organized into modules [also called components or compartments; 4–11]. A module is a subset of the populations in a community that interact mostly among each other and less frequently with populations outside the module. Many studies have concentrated on the structure of bipartite interactions [e.g., 12–15]. A bipartite community is one with two distinct levels with no omnivory, intraguild or intra level interactions and include pollinator and herbivore communities. Recent examples of non-bipartite modularity include Leaper et al. [16], who used species clustering to describe species modules in animal communities associated with coral reefs (which they called archetypes) and Cordero and Jackson [17], who used correlation analysis to identify modules in freshwater fish communities.

Ordinations have been commonly used to simplify terrestrial arthropod communities and reveal underlying community organization. Many of these studies have used constrained ordinations, which seek to understand how the arthropod community may be related to exogenous abiotic and biotic factors [e.g., 18–21]. Unconstrained ordination, relying on NMDS, has also been used frequently on terrestrial arthropod communities [e.g., 22–24] and in fewer cases, unconstrained ordination has been used to demonstrate modularity in a plant-arthropod herbivore community [25] or lack of modularity in an ant-food resource community [26]. However, these analyses have focused on bipartite communities with only two trophic levels and no intraguild interactions. To our knowledge, unconstrained ordination has not been used to look for modularity in more complex arthropod communities involving herbivores and natural enemies.

We used model-based unconstrained ordination coupled with randomization to test an hypothesis about the structure of the macroinvertebrate community associated with an annual agroecosystem in tropical Brazil. Annual agroecosystems characteristically have frequent, periodic major disturbances, such as tillage for field preparation, weed control, harvesting and post-harvest residue destruction. These disturbances disrupt biotic interactions and can result in a random community structure because there is insufficient time for the community to organize into strongly interacting multi-species modules. In this case, species population dynamics would be relatively independent of most of the other species in the community and populations could be studied in isolation without considering other populations. An alternative hypothesis is that colonization dynamics are rapid [27, 28] and the community quickly organizes into a repeatable structure as the annual crop develops. We test if the community becomes organized into repeatable modules or if it remains a random assemblage of species. Specifically, as suggested by [4], the macroinvertebrate community may become organized into modules around pest herbivores and their natural enemies.

## Methods

### Sampling

*Brassica oleracea* production fields at six organic vegetable farms in the Distrito Federal, Brazil were examined during 2017 and 2018: 15°39’06"S 48°06’33"W; 15°39’00"S 48°12’01"W; 15°36’43"S 48°04’43"W; 15°33’54"S 48°01’48"W; 15°44’18"S 47°39’13"W; and 15°45’44"S 47°38’29"W. Data were collected under authorization numbers SISBIO 36950 and IBAMA 02001.008598/2012-42. We focused on organic farms to avoid complications associated with pesticide use and none of the farmers used pesticides. Crops were grown according to recommendations for organic production [29]. Within a field in each farm, an area of about 200 m^2^ was designated as the sample area. Invertebrates were sampled at each farm every two weeks during the dry season (May to September, 10 sample dates total), because aphid populations, which are important pests of brassicas, are more abundant during the dry season. Each sample comprised a count of all invertebrates on 30 plants by visual inspection. All invertebrates were identified in the field to the lowest feasible taxonomic level based on pictorial keys and experts in the field. When aphids were sparse, we counted all aphids, but when they reached very high numbers, we estimated the total population by subsampling. We sampled 30 plants/sample time × 10 sample times/farm × 6 farms/year × 2 years = 3600 plants. For each sample date for each farm in each year, the total count of each taxon was calculated.

We studied the *B*. *oleracea* agroecosystem because it is a common vegetable crop worldwide, encompassing broccoli, cauliflower, brussels sprouts, collard greens, kale, and kohlrabi. It has a well-known macroinvertebrate fauna, which in our study area includes as herbivores, the lepidoptera *Plutella xylostella* (diamond-backed moth), *Trichoplusia ni* (cabbage looper) and *Ascia monuste* (great southern white), the aleyrodid *Bemisia tabaci* (silverleaf or sweet potato whitefly), the aphidids *Brevicoryne brassicae* (cabbage aphid), *Lipaphis pseudobrassicae* (turnip aphid) and *Myzus persicae* (green peach aphid), the polyphagous coleoptera *Diabrotica speciosa* (cucurbit beetle) and *Lagria villosa* (*bicho capixaba*) and some polyphagous Orthoptera, Diptera and Hemiptera.

We sampled the 36 most abundant macroinvertebrate populations. These included the herbivores *B*. *brassicae* + *L*. *pseudobrassicae*, *M*. *persicae*, *Uroleucon* spp. (Aphididae), aphid alatae, leafminers (Diptera), Lepidoptera eggs, Noctuidae (including *T*. *ni*), *P*. *xylostella* immatures, *A*. *monuste* immatures, Hemiptera herbivores (primarily Pentatomidae), *B*. *tabaci*, Galerucinae (including *D*. *speciosa*), *L*. *villosa*, *Astylus variegatus* (maize pest), Orthoptera, and Gastropoda. Natural enemies included the predators coccinellid eggs, *Harmonia axyridis* adults, *Ha*. *axyridis* immatures, *Cycloneda sanguinea* adults, *C*. *sanguinea* immatures, *Hippodamia convergens* adults, *Hi*. *convergens* immatures, *Eriopis connexa* adults, *E*. *connexa* immatures, spiders, Dolichopodidae (long-legged flies, all adults), Syrphidae (hoverflies, larvae and adults), *Chrysoperla externa* (green lacewings, nearly all eggs), Hemiptera predators, and Staphylinidae. Parasitoids were sampled as aphid mummies, microhymenoptera (nearly all aphid parasitoids) and large parasitoids (mostly lepidopteran parasitoids). Muscoidea (flies) and Formicidae (ants) were also sampled.

### Community analysis

We used model-based unconstrained ordination to extract the organization of the macroinvertebrate community [30]. These model-based methods have several advantages over the distance-based methods [31], such as non-metric multidimensional scaling (NMDS), especially for the analysis of macroinvertebrate counts. Distance-based methods transform counts, typically with the square root transform. However, as arthropod counts are rarely Poisson distributed, the square root transform actually gives greater weight in a NMDS to the most highly over-dispersed populations and does not provide a clean signal of the effects only of community composition. Model-based methods can explicitly model population abundance for each population in the community, which stabilizes variance, enabling the detection of a clean signal of community composition. In addition, model-based methods can use residual analysis to check how well the model fits the data and can use maximum likelihood tools, such as AIC and BIC, to assist with model selection. The disadvantage of model-based methods is that the number of parameters estimated can be substantially greater than for distance-based methods [32]. In our case, which is a two-dimensional ordination with 36 populations and 120 samples, NMDS would fit 240 parameters, while the model-based method would fit 504 parameters with separate negative binomial responses for each population. This gives an average of 8.6 observations per parameter for the model-based method with our data.

We conducted the model-based unconstrained ordination using the R package *boral* [33]. We assumed a negative binomial mean-variance relation for our count data and evaluated latent variable models (LVM) with 0–4 latent variables, with fixed or random sample effects, and with samples nested within a random farm × year effect or no farm specification. To evaluate convergence of the MCMC Gibbs sampler, we considered the Gewenke convergence criterion and adjusted the *p*-values using the Holm-Bonferroni method [34] as suggested and implemented by the authors of *boral*. When sample effects are fixed, the LVM is an analysis of community composition, as the fixed effects remove the effect of variation in total abundance among samples. When sample effects are random, the LVM returns an analysis allowing for quadratic population responses [35], which means the populations can increase and then decrease with time. Nesting within the farms separates the sample effect into a farm effect and a within farm sample effect and allows for evaluation of spatial variation among farms in composition. We used AIC to help with model selection but based the final model selection mainly on biological criteria as recommended by the package authors [33]. We selected the LMV with 2 latent variables, fixed samples with nesting of farms (S1 Table), which is given as

$$g\left(\mu_{ij}\right)=\alpha_{i}+\beta_{0j}+u_{i}^{t}\theta_{j}$$ (1)

where the mean response of sample *i* and species *j*, *μ*_*ij*_, is regressed via the log link, *g*, with negative binomial error, on *α*_*i*_ which are the fixed sample effects and are hierarchically estimated as a farm × year random effect with fixed sample effects within farms × years, *β*_0*j*_ which are the response specific intercepts, and the product of *u*_*i*_ which is a matrix of the vectors of latent variables and *θ*_*j*_ which are response-specific coefficients relating to these latent variables. Specifically, we used the *boral* function, boral(y = abund, family = "negative.binomial", lv.control = list(num.lv = 2), row.eff = "fixed", raneff.ids = matrix(rep(1:12, each = 10), ncol = 1)).

### Community randomization

We randomized the data to have 2–5 modules using a customization of the R script GenDataSample, which we called GenDataSampleMod (S1 File), from the *RGenData* package as recommended by the author [36]. We defined modules to have greater association within modules and lower association among modules. We generated random modules by ordering populations from most abundant to least abundant and assigning the most abundant population to module 1, the second most abundant to module 2 and so on for the required number of modules and repeating the process until all populations were assigned to a module. This was done to ensure that population abundance would have minimal effect on the random modules, which would be uncontrolled if we had used a randomized assignment process. We then randomized the data within modules to have within module correlations of 0.4 and between module correlations of 0, to examine how the 2-factor latent variable model would characterize the modules in the community. Subsequently, we randomized data for the two-module randomization to have a more realistic correlation structure that was representative of the original data with within module correlations of 0.25 and between module correlations of 0.02. We conducted 10 randomization trials for each condition using a custom R script (S1 File) with GenDataSampleMod and fitted model Eq (1) to these data.

We also randomized the data to generate a dataset with reduced correlation among the populations to simulated randomly organized communities using another custom R script (S1 File) and fitted model Eq (1) to the random data. We independently randomized counts among samples for each population preserving the population counts but not the summed counts of the populations in each sample. We randomized the original data 500,000 times, calculated the 1260 *p*-values of the Pearson correlation matrix for each randomization, and selected the randomization with *p*-values that were distributed most closely to a uniform distribution as estimated by the Kolmogorov-Smirnov distribution test. A uniform distribution of *p*-values is expected when *p*-values are randomly distributed and the community is randomly assembled.

We tested if spatial variation among the farms affected LV1, LV2 and *β*_0*j*_, which is the average *ln* population counts for each sample and is indicative of variation in total abundance of the sample. These analyses evaluate the potential role of the species pool surrounding the farms on the community composition. We used lmer in the *lme4* package [37], treating the observation periods (Time) within farms and years as repeated measures, as follows:

Response~Time+(1|Farm)+(1|Year)

where Response is either LV1, LV2 or *β*_0*j*_. We estimated the significance of the random effect of farm using the likelihood ratio test (2 × difference in log-likelihood of nested models), which is a conservative test.

### Statistical analysis

We compared the latent variable models of the observed data against the hypothesis of random assemblage and an assemblage of two modules assuming that the latent variable models were bivariate distributions. We compared the statistical similarity of the two marginal distributions of the latent variables first with the Shapiro-Wilk normality test and then, based on the results of [38], we used Fisher’s method for combining *p*-values to compare the models as bivariate normal distributions. We also compared the Pearson correlations between latent variables for the three models using the Fisher transformation to *z*-values.

## Results

### Ordination model

**Fig 1:**
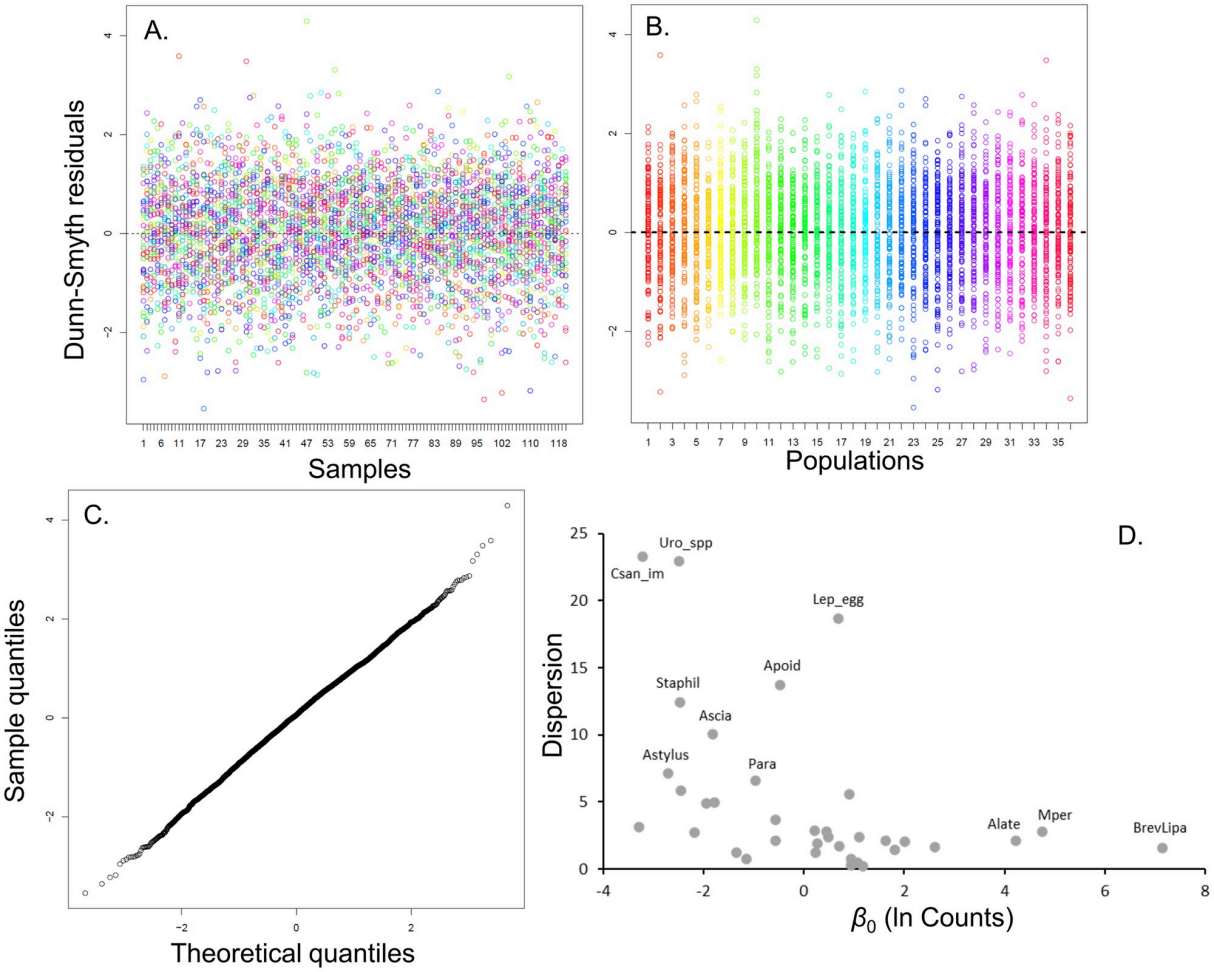
Diagnostics for selected boral model with two latent variables. (A) Residual plot for samples (colors are different populations); (B) Residual plot for populations; (C) Q-Q plot; (D) Population dispersion for negative binomial versus population means.

The *boral* model with two latent variables (LV1 and LV2) and negative binomial population responses fit the data well. Residual analysis of the fitted samples and the fitted populations showed uniform, symmetric distributions (Fig 1A and 1B) and the Q-Q plot was virtually linear with the exception of a few samples in the tails of the distribution (Fig 1C). The population-specific negative binomial response model was essential (Fig 1D), as there was considerable variation in dispersion among taxa and overdispersion in nearly all taxa.

**Fig 2:**
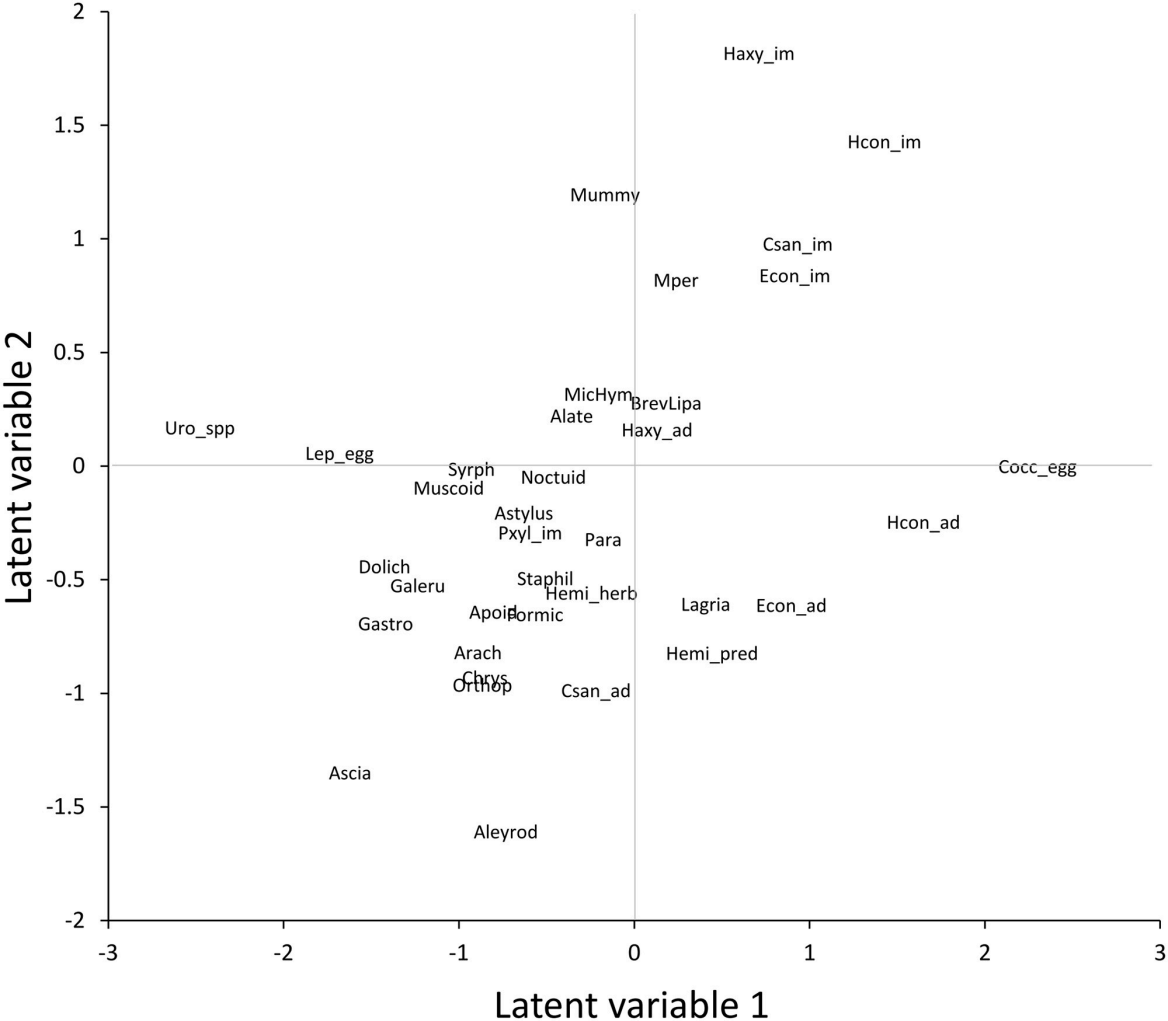
Unconstrained ordination of original data. Axes are latent variables from the selected boral model.

The unconstrained ordination (Fig 2) indicated that the brassica aphids and coccinellids separated from other populations by having positive values on LV1, while the lepidopteran, aleyrodid and other aphidid (*Uroleucon* spp.) herbivores and dolichopodid, syrphid, chrysopid, staphylinid and Arachnida predators had negative values. None of the populations with negative LV1 values had high population counts in the brassica fields. LV2 had immature coccinellids, aphid mummies, microhymenopteran (primarily aphid parasitiods), and the brassica aphids *M*. *persicae*, *B*. *brassicae* + *L*. *pseudobrassicae* with positive values. All other taxa had negative or close to 0 values on LV2. Overall, the ordination suggested one module comprising brassica aphids, aphid parasitoids (mummies and microhymenoptera) and all of the aphidophagous coccinellids, except for *C*. *sanguinea* adults, and another module comprising lepidoptera and aleyrodids along with dolichopodid, syrphid, chrysopid, staphylinid and Arachnida predators. Unexpectedly, ants (“Formic” in Fig 2) were not associated with the common aphids, suggesting that they were not functioning mainly as aphid mutualists.

### Randomized data into community components

**Fig 3:**
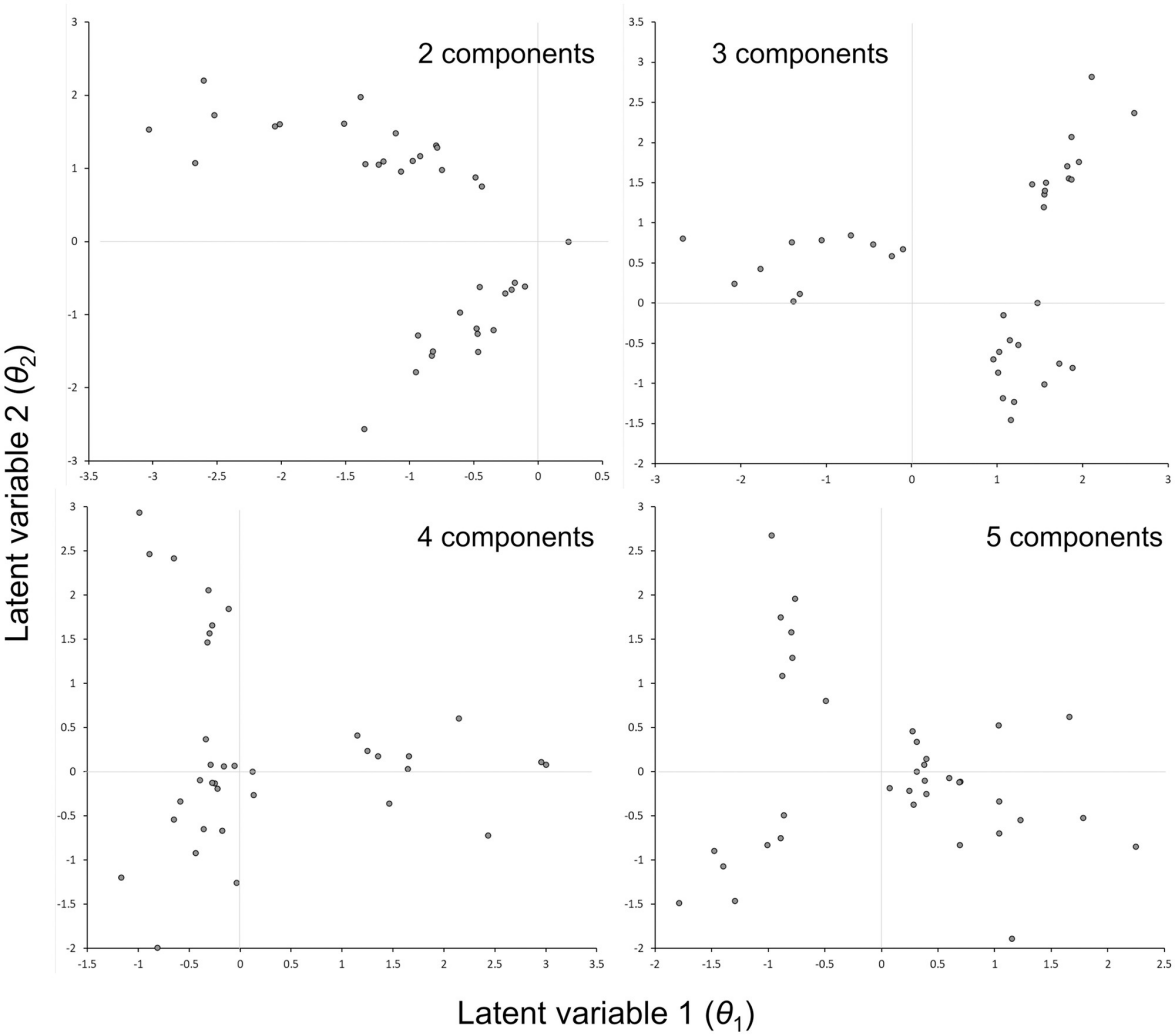
Unconstrained ordinations of randomized data. Randomizations have 2–5 modules with target Pearson correlation within modules = 0.40 and between modules = 0.00.

The randomizations of the community with 2–5 modules and target correlations of 0.4 within modules and 0.0 between modules generated data with the desired distributions of correlations (S1 Fig). Unconstrained ordinations with two latent variables on the randomized data with 2 or 3 modules readily distinguished the 2 or 3 modules (Fig 3). The 2-module ordination had two distinct modules but showed evidence of the arch effect that sometimes occurs in ordinations. The 3-module ordination showed three distinct clusters of populations, similar to a 3-leaf clover. When randomized with 4 or 5 modules, however, the 2-dimensional ordination for these additional modules was visually similar to the 3-module ordination. We repeated this analysis for 2 and 3 modules with a lower within module target correlation = 0.25 (S2 Fig). With the lower within module correlation, 2 and 3 modules were still readily distinguished, and the arch effect in the 2-module ordination completely disappeared.

### Hypothesis tests

**Fig 4:**
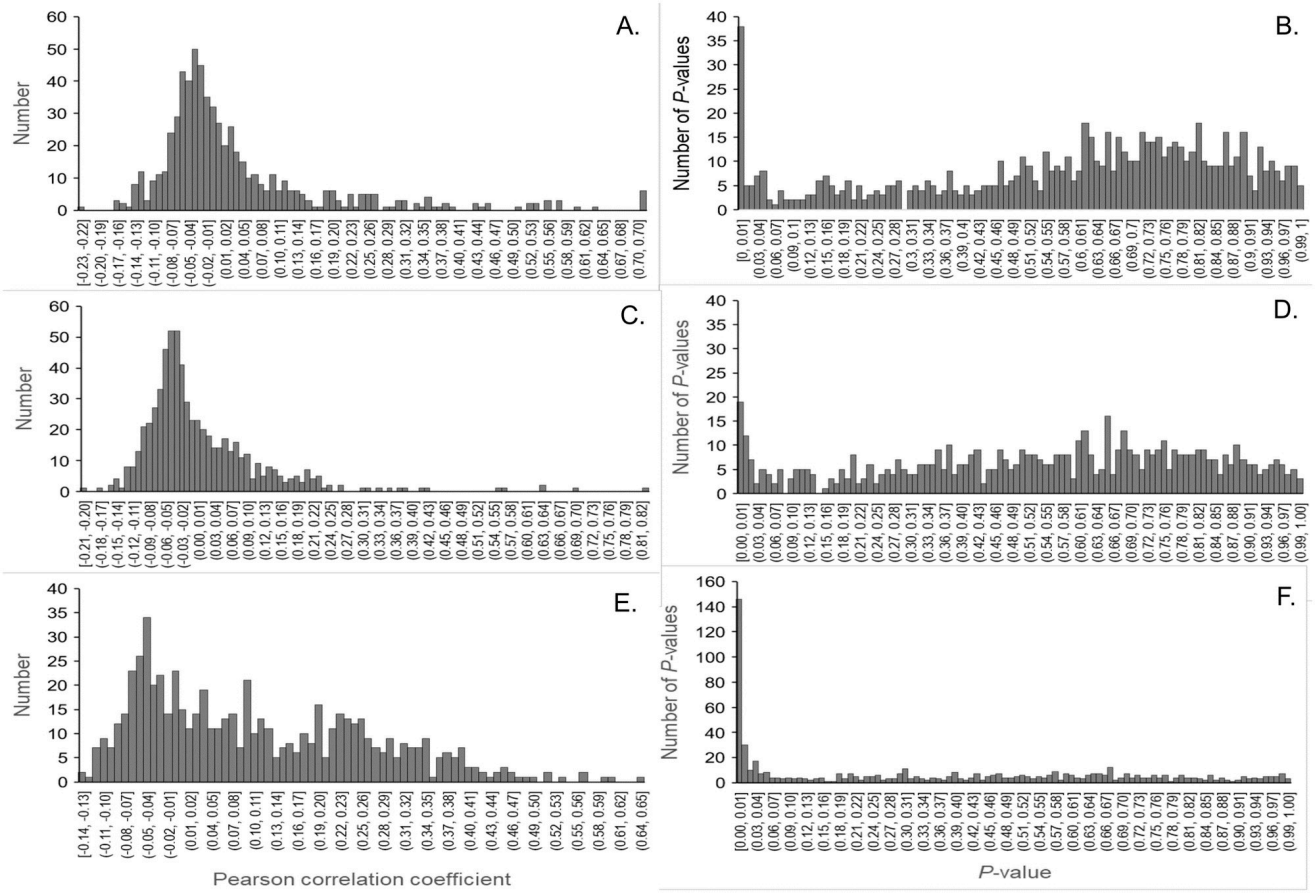
Histograms of all possible Pearson correlation coefficients and associated *p*-values for original data. (A, B), completely randomized data (C, D), and randomized data with two components (E, F).

As the ordination of the actual data did not resemble a 3-module ordination, we used the randomized 2-module ordinations for hypothesis testing with the more realistic target correlations of 0.25 within modules and 0.02 between modules. The between module correlation allows for some overlap of populations among modules. A higher between module correlation would blend the two modules until modules could no longer be distinguished. The correlation structure and distribution of *p*-values for the original data, the randomized 2-module data, and the completely randomized data are shown in Fig 4. The original data had correlation coefficients distributed around -0.03 with a long positive tail with some clustering around 0.10 and 0.25. The *p*-values had a large number with values <0.01 and another peak around 0.75. The completely randomized data had correlation coefficients distributed around -0.03 and had a positive tail that was lighter than the original data. The *p*-values were not uniformly distributed, but the number with values <0.01 was halved and there was only a slight peak around 0.65. Perhaps an additional process based on simulated annealing would have produced a data set with a uniform distribution of *p*-values, but this would no longer be a random data set. Clearly, the present completely randomized data have *p*-values more uniformly distributed than the original data. The randomized 2-module data had correlation coefficients with peaks around -0.04 and 0.25. Thus, the data randomizations approximated a completely random community and a random 2-module community.

**Fig 5:**
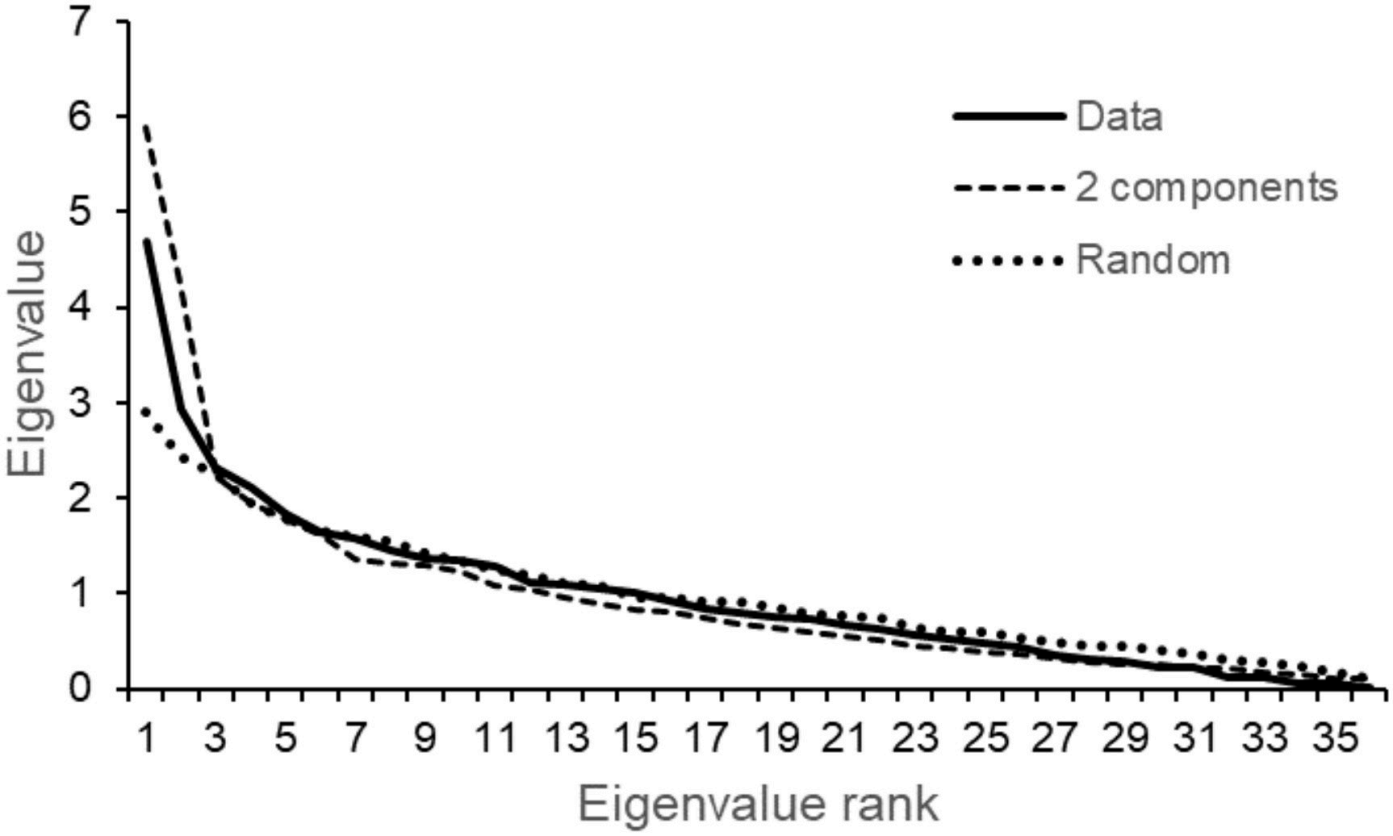
Eigenvalue plot. Original data (Data), randomized 2-component data (2 modules) and completely randomized data (Random).

The eigenvalues of the correlation matrices for the three data sets (original, random and randomized 2-module) showed marked deviations for the first two eigenvalues, and close similarity for the remaining eigenvalues (Fig 5). This implies that two latent variables should capture most of the differences among the data sets and their respective *boral* models. The eigenvalue profile of the original data is intermediate to the other two but more similar to the randomized 2-module data than the completely randomized data.

**Fig 6:**
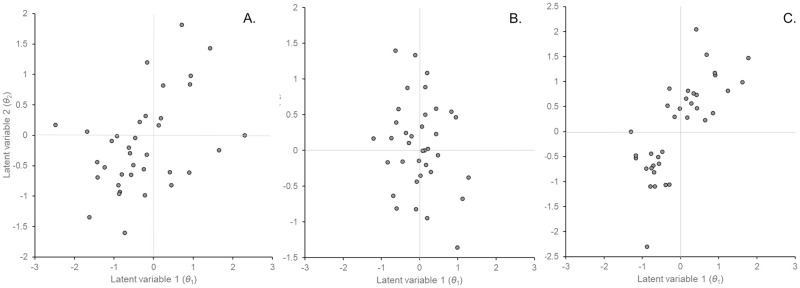
Unconstrained ordinations, (A) original data, (B) completely randomized data and (C) randomized 2-component data using boral with fixed site effects and negative binomial errors.

The ordinations comparing the original data with the randomized 2-module data and the completely randomized data are shown in Fig 6. Visually, the ordination of the original data (Fig 6A) appears different from the ordination of the completely randomized data (Fig 6B) and similar to the ordination of the randomized 2-module data (Fig 6C). Normality tests of the marginal distributions of the three ordinations did not reject the hypothesis that they were normally distributed (Table 1), and the lowest *p*-value was 0.1920. Therefore, we considered the ordinations to approximate bivariate normal distributions. We found that the marginal distributions of the original data were significantly different from those of the completely randomized data (*p* = 0.0031), while they were not statistically different from those of the randomized 2-module data (*p* = 0.1496, Table 1). The difference from the completely randomized data was due to the difference in the variance in LV1, which was significantly smaller for the completely randomized data. In other words, LV1 captured significant variation in community composition in the original data. Similarly, the correlation between the LVs for the original data was significantly different from the completely randomized data (*p* = 0.0175, Table 2), but was not significantly different from the randomized 2-module data (*p* = 0.3099). Thus, the community composition of original data is not random, but is consistent with the existence of two modules in the community.

**Table 1:** Marginal distributions of latent variables (LV1 and LV2) and comparison of original data with randomized data and two-factor random data.

		Marginal distribution		Normality test		Mean comparison		Variance comparison		Fisher’s method	
		*μ*	*s* ^2^	*W*	*p*-value	*t*_35_	*p*-value	*F*_35,35_	*p*-value	χ^2^_8_	*p*-value
Original data	LV1	-0.274	1.008	0.98066	0.7672						
	LV2	-0.156	0.583	0.95925	0.2038						
Randomized data	LV1	0.010	0.331	0.97632	0.6204	1.473	0.1496	3.049	0.0014	23.230	0.0031
	LV2	0.074	0.394	0.99027	0.9848	1.394	0.1721	1.480	0.2513		
2-factor data	LV1	-0.052	0.624	0.95842	0.1920	1.039	0.3059	1.616	0.1605	12.037	0.1496
	LV2	0.100	0.877	0.97038	0.4361	1.270	0.2123	1.502	0.2334		

Marginal distributions show sample means and variances. Normality test with Shapiro-Wilk test (*W*), mean comparison with t-test using pooled variances, variance comparison using ratio of variances with 2-tailed *p*-value, and Fisher’s method for combining *p*-values.

**Table 2:** Independence tests of latent variables.

	*r*	H_0_(*r* = 0)		H_0_(*r*_orig_ = *r*_other_)	
		*z*	*p*-value	*z*_obs_	*p*-value
Original data	0.4198	2.283	0.0224		
Randomized data	-0.1899	-1.078	0.2811	2.377	0.0175
2-factor data	0.7562	3.719	0.0002	-1.015	0.3099

H_0_(*r* = 0) and comparison of correlation between latent variables with original data, H_0_(*r*_orig_ = *r*_other_). Significant tests for the Pearson correlation coefficients use Fisher’s *z*-transformation.

### Temporal change and spatial effects

**Fig 7:**
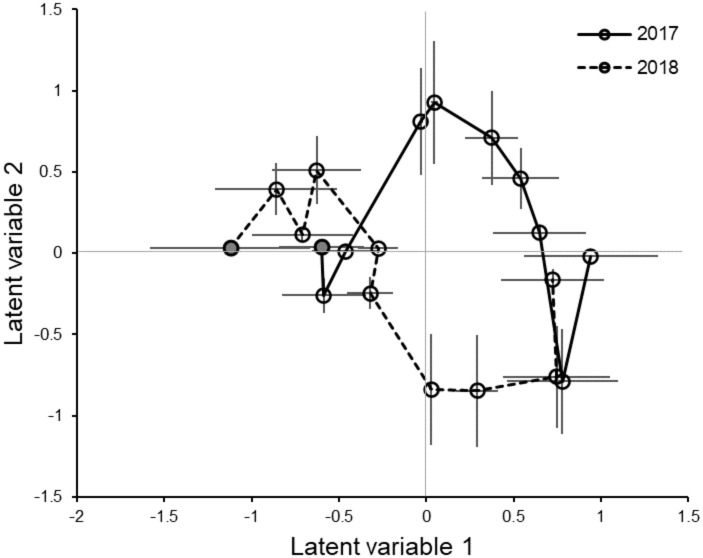
Temporal change in community composition in 2017 and 2018. Points are mean of 6 farms with SE. First sample in the season is shown with the gray circle. Each subsequent sample was taken at 2-week intervals.

We examined the temporal change in community composition by graphing the centroids of samples collected during the same week in each year. In both years, community composition changed gradually from negative LV1 values to positive LV1 values (Fig 7). Biologically this means that the composition shifted from a lepidopteran/aleyrodid community module with their associated predators to a brassica aphid community module with their parasitoids and associated coccinellid predators. This pattern was consistent among farms and between years (S3 Fig). The major difference between the years was that in 2017 the dynamic was strongly driven by the brassica aphids and associated natural enemies, while this did not occur as strongly in 2018.

The random effect of farm was significant for LV1, LV2 and *β*_0*j*_ (Table 3). The effects on the latent variables indicated that the composition of the community varied among the farms, which suggests that the surrounding species pool affected community composition. The effect on *β*_0*j*_ indicated that the total community abundance also varied among farms.

**Table 3:** Significance tests of the random effect of farm.

	Farm
LV1	53.629 (2.42 E-13)
LV2	14.676 (1.27 E-4)
*β*_0*j*_	7.834 (0.0051)

Likelihood ratio test with *χ*^2^ and *p*-value (in parentheses) for *df* = 1. LV is latent variable and *β*_0*j*_ is the average ln count for each sample.

## Discussion

### Community composition

We hypothesized that the frequent periodic large disturbances associated with planting, weeding and harvest in the organic brassica system in central Brazil may preclude the development of strong interactions among populations, resulting in a random community structure in which pest species could be studied in isolation. Our results rejected this hypothesis. Instead, we found evidence supporting the hypothesis that the community quickly organized into repeatable modules. The macroinvertebrate community associated with organic brassica farms was structured into two multi-population modules organized around different herbivores and associated natural enemies. One module comprised the brassica aphids and the aphidophagous natural enemies, and the other module comprised lepidopteran and aleyrodid herbivores and associated natural enemies. Model-based unconstrained ordination revealed that two latent variables were sufficient to characterize the major variation in community composition. This was shown both by model selection using AIC and by the convergence of the higher order eigenvalues for the original data, the completely randomized data and the random 2-module data. Comparing the ordination of the original data with that of the completely randomized data, we determined that the first latent variable accounted for more variation than at random and was the origin of the non-random organization of community composition. When comparing the ordination with several alternative hypotheses with the community organized into 2–5 modules, we found that the observed community composition was not significantly different from the two-module hypothesis and differed from the 3- to 5-module hypotheses. In addition, the repeatable seasonal change of the arthropod community composition in the six farms and two years supported the existence of two community modules. During the growing season, there was a gradual shift from a lepidopteran/aleyrodid community module with associated natural enemies to a brassica aphid community module with aphid parasitoids and coccinellid predators.

These results provide strong support for the life systems approach [3] for analyzing arthropod herbivore pests in agroecosystems, which focuses on the major herbivore pests and their associated natural enemies. Most recent work on macroinvertebrate communities associated with annual agroecosystems in the neotropics has focused on a few pest species in isolation [e.g., 39–42]. These investigations begged the question if a broader understanding of community organization is essential for understanding pest biology in annual tropical agriculture. In contrast to these narrowly focused studies, our study and Malaquias et al. [43] sampled the entire aboveground macroinvertebrate community and used dimensional-reduction methods to suggest that a simplified understanding of the community was sufficient. However, only our study simplified the populations into modules. More broadly, our study and [43] together suggest that a reduced, life systems focus can reveal important community dynamics in tropical annual cropping systems. Moreover, this result implies that sustainable pest management solutions can be found without having to consider all potential interactions in the macroinvertebrate community.

### Broader implications

Community organization is widely recognized to result from the filter of colonization [e.g., 44], followed by the local abiotic and biotic filters that are typically historically contingent [e.g., 45]. Our results have emphasized that the local filters are important and that biotic interactions likely structured the macroinvertebrate community associated with organic brassica in central Brazil. We also recognized the stochastic nature of colonization and found that the communities were significantly influenced by the variation in the species pools surrounding each farm. This biogeographic contingency is an important factor influencing community organization and has been suggested to sometimes swamp the effects of the local abiotic and biotic processes [e.g., 46]. In our case, despite the significant stochasticity, a repeatable signal of community structure was detected. Thus, even in highly disturbed environments, such as annual agriculture, local processes can dominate, leading to regularity in community structure.

## Supporting information

S1 Fig Histograms of Pearson correlation coefficients. For randomized data with 2–5 modules with target Pearson correlations within modules = 0.40 and between modules = 0.00.
(PDF)

S2 Fig Unconstrained ordinations of randomized data with 2–3 modules. For target Pearson correlation within modules = 0.25 and between modules = 0.00.
(PDF)

S3 Fig Temporal change in community composition in six farms over two years (2017 and 2018). Farms are numbered 1–6. Each dot is one sample time and the first sample time of the season is indicated by the large dot. Community composition starts with negative values for latent variable 1 which gradually turn more positive (except for 2017–2).
(PDF)

S1 File Custom R scripts. (A) customization of GenDataSample, which is remaned GenDataSampleMod; (B) randomization trials and model fitting; (C) simulated randomly organized communities and model fitting.
(PDF)

S1 Table AIC calculated at parameter medians^1^. Bold are minimum AIC for each row.
(PDF)
