# Supplementary material for: Organization of the macroinvertebrate community in a tropical annual agroecosystem into modules
Source: PLoS One. 2023 Aug 3;18(8):e0289103. doi: 10.1371/journal.pone.0289103 (PMC10399829; doi:10.1371/journal.pone.0289103)
Supplement: S1 Fig — For randomized data with 2–5 modules with target Pearson correlations within modules = 0.40 and between modules = 0.00. (PDF) [file pone.0289103.s001.pdf]

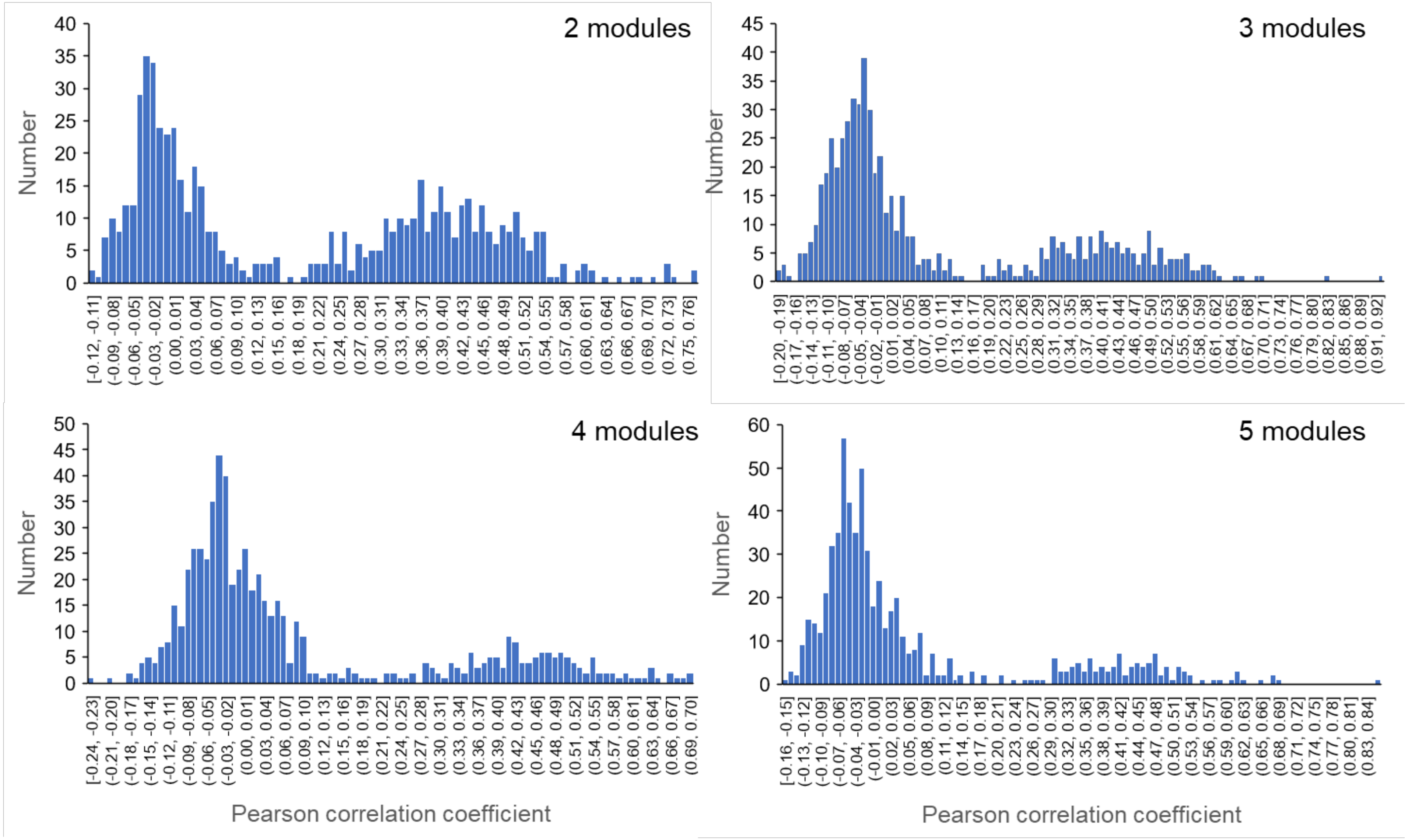

S1 Fig. Histograms of Pearson correlation coefficients of randomized data. For 2-5 modules with target Pearson correlations within modules = 0.40 and between modules = 0.00.
