# Supplementary material for: Organization of the macroinvertebrate community in a tropical annual agroecosystem into modules
Source: PLoS One. 2023 Aug 3;18(8):e0289103. doi: 10.1371/journal.pone.0289103 (PMC10399829; doi:10.1371/journal.pone.0289103)
Supplement: S2 Fig — For target Pearson correlation within modules = 0.25 and between modules = 0.00. (PDF) [file pone.0289103.s002.pdf]

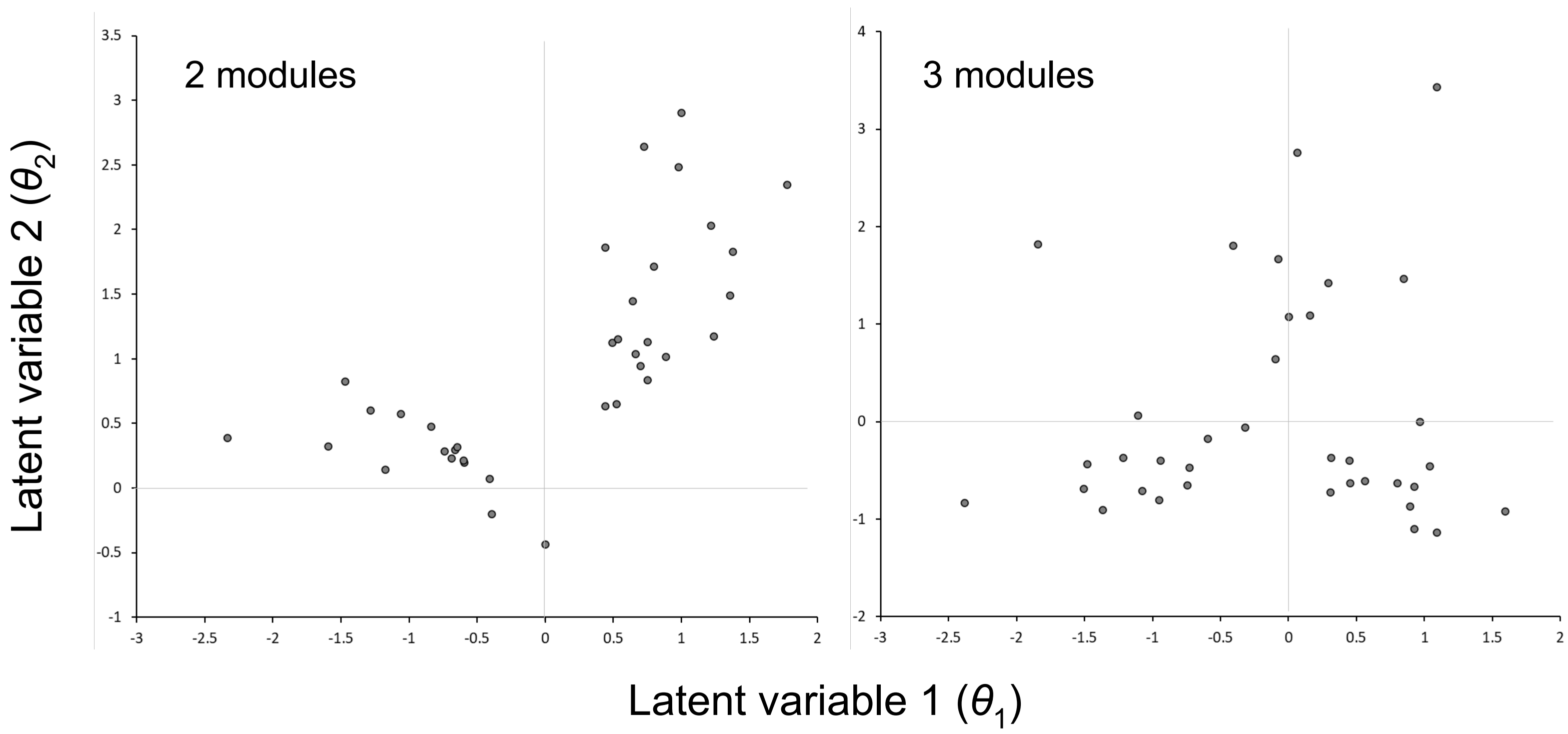

S2 Fig. Unconstrained ordinations of randomized data. For 2-3 modules with target Pearson correlation within modules = 0.25 and between modules = 0.00.
