# Supplementary material for: Organization of the macroinvertebrate community in a tropical annual agroecosystem into modules
Source: PLoS One. 2023 Aug 3;18(8):e0289103. doi: 10.1371/journal.pone.0289103 (PMC10399829; doi:10.1371/journal.pone.0289103)
Supplement: S3 Fig — Farms are numbered 1–6. Each dot is one sample time and the first sample time of the season is indicated by the large dot. Community composition starts with negative values for latent variable 1 which gradually turn more positive (except for 2017–2). (PDF) [file pone.0289103.s003.pdf]

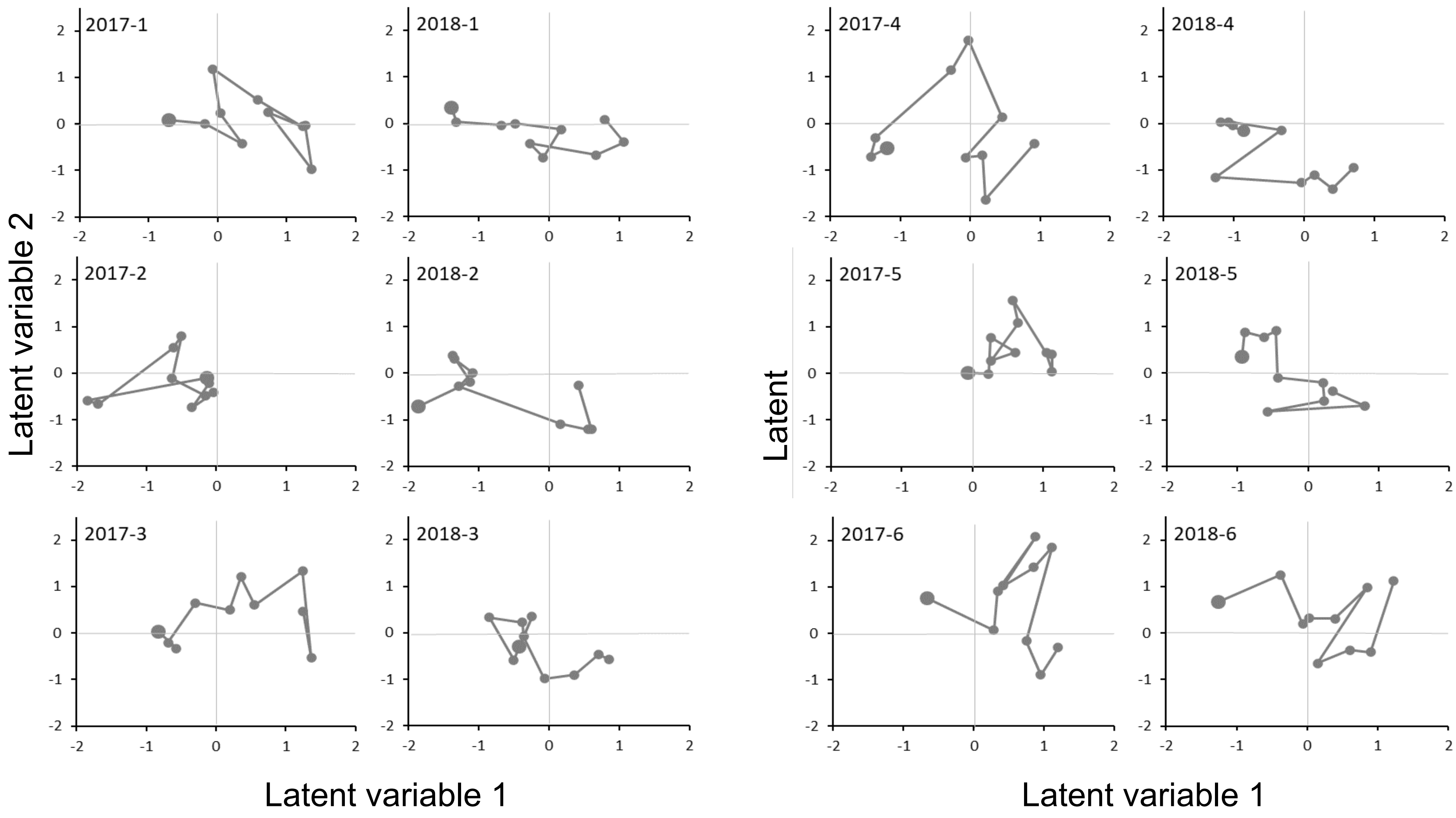


**S3 Fig. Temporal change in community composition in six farms over two years (2017 and 2018).** Farms are numbered 1-6. Each dot is one sample time and the first sample time is indicated by the large dot. Community composition starts with negative values for latent variable 1 which gradually turn more positive (except for 2017-2).
