# Supplementary material for: Organization of the macroinvertebrate community in a tropical annual agroecosystem into modules
Source: PLoS One. 2023 Aug 3;18(8):e0289103. doi: 10.1371/journal.pone.0289103 (PMC10399829; doi:10.1371/journal.pone.0289103)
Supplement: S1 File — (A) customization of GenDataSample, which is remaned GenDataSampleMod; (B) randomization trials and model fitting; (C) simulated randomly organized communities and model fitting. (PDF) [file pone.0289103.s004.pdf]

S1 File. Custom R scripts. (A) customization of GenDataSample, which is remaned GenDataSampleMod; (B) randomization trials and model fitting; (C) simulated simulated randomly organized communities and model fitting

```
## (A) customization of GenDataSample, which is remaned GenDataSampleMod
################################################################################
################################################################################
################################################################################
##### Yellow highlights indicate changes to GenDataSample
GenDataSampleMod <- function(supplied.data, target.corr, n.factors = 0, max.trials = 5, #added target.corr
                   initial.multiplier = 1, corr.type = "pearson", seed = 0)
# Bootstraps each variable's score distribution from a supplied data set.
#
# Args:
#   supplied.data    : Data supplied by user. An I sample (rows) by J population (columns) matrix or dataframe
#   target.corr      : the target correlation matrix, square symmetric J x J; must be supplied
#   n.factors        : Number of factors (scalar, default is 0). #cannot be default, must specify n.factors; if not
#                      provided script may crash if Random.Data draws a vector of 0's
#   max.trials       : Maximum number of trials (scalar, default is 5).
#   initial.multipiler : Value of initial multiplier (scalar, default is 1).
#   corr.type        : Type of correlation (character, default is "pearson",
#                  user can also call "spearman").
#   seed             : seed value (scalar, default is 0).
#
# Returns:
#   data : Sample of data
################################################################################
  set.seed(seed)
  n.cases <- dim(supplied.data)[1]
  n.variables <- dim(supplied.data)[2]
  data <- matrix(0, nrow = n.cases, ncol = n.variables)
  distribution.matrix <- matrix(0, nrow = n.cases, ncol = n.variables)
  iteration.count <- 0
  best.rmsr.corr <- 1
  trial.counter <- 0
  if (seed != 0)
    set.seed(seed) # If user specified a nonzero seed, set it

    # Modify this block of the program, as needed, to generate the desired distribution(s).
  for (i in 1:n.variables)
    distribution.matrix[, i] <- sort(sample(supplied.data[, i], n.cases, replace = TRUE))

  target.correlation <- target.corr   # original was cor(supplied.data)
  intermediate.correlation <- target.correlation

#  if (n.factors == 0) { #This section is commented out to avoid crashing from 0s in a sparse data matrix
#    eigenvalues.observed <- eigen(intermediate.correlation)$values
```

```
#    eigenvalues.random <- matrix(0, nrow = 100, ncol = n.variables)
#    random.data <- matrix(0, nrow = n.cases, ncol = n.variables)
#    for (i in 1:100) {
#      for (j in 1:n.variables)
#        random.data[, j] <- sample(distribution.matrix[, j], size = n.cases,
#        replace = TRUE)
#      eigenvalues.random[i, ] <- eigen(cor(random.data))$values
#    } #end for i
#    eigenvalues.random <- apply(eigenvalues.random, 2, mean)
#    # calculate mean eigenvalue for each factor
#    n.factors <- max(1, sum(eigenvalues.observed > eigenvalues.random))
#  } #end if n.factors==0

  shared.components <- matrix(rnorm(n.cases * n.factors, 0, 1), nrow = n.cases, ncol = n.factors)
  unique.components <- matrix(rnorm(n.cases * n.variables, 0, 1), nrow = n.cases, ncol = n.variables)
  shared.load <- matrix(0, nrow = n.variables, ncol = n.factors)
  unique.load <- matrix(0, nrow = n.variables, ncol = 1)

  while (trial.counter < max.trials) {
    iteration.count <- iteration.count + 1
    factor.analysis <- FactorAnalysis(intermediate.correlation, corr.matrix = TRUE, max.iteration = 50,
             n.factors, corr.type)
    if (n.factors == 1) {
      shared.load[, 1] <- factor.analysis$loadings
    } else {
      shared.load <- factor.analysis$loadings
    } # end if n.factors==1
    shared.load[shared.load > 1] <- 1
    shared.load[shared.load < -1] <- -1
    if (shared.load[1, 1] < 0)  shared.load <- shared.load * -1
    shared.load.sq <- shared.load * shared.load
    for (i in 1:n.variables)
      if (sum(shared.load.sq[i, ]) < 1) {
        unique.load[i, 1] <- (1 - sum(shared.load.sq[i, ]))
      } else {
        unique.load[i, 1] <- 0
      } # end if sum(shared.load.sq)
    unique.load <- sqrt(unique.load)
    for (i in 1:n.variables)
      data[, i] <- (shared.components %*% t(shared.load))[, i] + unique.components[, i] * unique.load[i, 1]
    for (i in 1:n.variables) {
      data <- data[sort.list(data[, i]), ]
      data[, i] <- distribution.matrix[, i]
    } # end for i in n.variables
    reproduced.corr <- cor(data)
    zero<- which(colSums(data)==0) #ensure that there are no NAs in corr mat.
    if(length(zero) > 0) for(x in 1:length(zero)) reproduced.corr[zero[x],zero[x]] <- 1
    reproduced.corr[is.na(reproduced.corr)] <- 0
```

```
    residual.corr <- target.correlation - reproduced.corr
    rmsr <- sqrt(sum(residual.corr[lower.tri(residual.corr)] * residual.corr[lower.tri(residual.corr)]) / (.5 *
          (n.variables * n.variables - n.variables)))
    if (rmsr < best.rmsr.corr) {
      best.rmsr.corr <- rmsr
      best.corr <- intermediate.correlation
      best.res <- residual.corr
      intermediate.correlation <- intermediate.correlation + initial.multiplier * residual.corr
      trial.counter <- 0
    } else {
      trial.counter <- trial.counter + 1
      current.multiplier <- initial.multiplier * .5 ^ trial.counter
      intermediate.correlation <- best.corr + current.multiplier * best.res
    } #end if rmsr < best.rmsr.corr
  } #end while trial.counter

  factor.analysis <- FactorAnalysis(best.corr, corr.matrix = TRUE, max.iteration = 50, n.factors, corr.type)
  if (n.factors == 1) {
    shared.load[, 1] <- factor.analysis$loadings
  } else {
    shared.load <- factor.analysis$loadings
  } #end if n.factors==1
  shared.load[shared.load > 1] <- 1
  shared.load[shared.load < -1] <- -1
  if (shared.load[1, 1] < 0)    shared.load <- shared.load * -1
  shared.load.sq <- shared.load * shared.load
  for (i in 1:n.variables)
    if (sum(shared.load.sq[i, ]) < 1) {
      unique.load[i, 1] <- (1 - sum(shared.load.sq[i, ]))
    } else {
      unique.load[i, 1] <- 0
    } #end if sum(shared.load.sq)
  unique.load <- sqrt(unique.load)
  for (i in 1:n.variables)
    data[, i] <- (shared.components %*% t(shared.load))[, i] + unique.components[, i] * unique.load[i, 1]
  data <- apply(data, 2, scale)
  for (i in 1:n.variables) {
    data <- data[sort.list(data[, i]), ]
    data[, i] <- distribution.matrix[, i]
  } #end for i in n.variables
  data <- data[sample(1:n.cases, n.cases, replace = FALSE), ]
  return(data)
}
#######################################################################################
#######################################################################################
```

(B) randomization trials and model fitting

```
#################################################################################
#################################################################################
#################################################################################
## create random data matrix with random correlations among species
setwd("C:/Users/User/Desktop/Debora work/Harmonia/Embrapa proposal/Pop Fluct")

library(tidyverse)
library(boral)
library(Hmisc)
abund <- as.matrix(read.csv("Counts_corrected.csv", header=T, stringsAsFactors = F))
rand_abund <- abund
KS <- 1
for(i in 1:500000) {
  for(j in 1:dim(abund)[2]) { rand_abund[,j] <- sample(abund[,j]) } #Randomize data for each population
  rand.corr <-rcorr(as.matrix(rand_abund), type="pearson") #Calculate correlations and p-values
  corr.p<- rand.corr$P[lower.tri(rand.corr$P)] #Remove p-values into a list
  ks <- ks.test(corr.p, punif)$statistic #Calculate Kolmogorov-Smirnov test for p-values against uniform
distribution
  if(ks<KS) {  #Save data with lowest KS test statistic
    Rand.Data <- rand_abund
    KS <- ks
    } #end if
  } #end for i
##Report and print best result; Rand.Data used in boral
KS
rand.corr <- rcorr(as.matrix(Rand.Data), type="pearson")
p.rand<- rand.corr$P[lower.tri(rand.corr$P)]
corr.rand <- rand.corr$r[lower.tri(rand.corr$r)]
eigen.rand <- eigen(rand.corr$r)$values
ks.test(p.rand,punif)
p.rand
corr.rand
eigen.rand
#################################################################################
#################################################################################
```

(C) simulated simulated randomly organized communities and model fitting

```
##########################################################################################
##########################################################################################
##########################################################################################
## create data matrix with specified correlations among populations and
## generate random data matrices with known number of groups and known within group correlations and
## known between group correlations (can be changed at yellow highlights), run boral for each simulation
setwd("C:/Users/User/Desktop/Debora work/Harmonia/Embrapa proposal/Pop Fluct")

library(tidyverse)
library(boral)
library(Hmisc)
library(RGenData)
source("GenDataSampleMod.R")

fac.vec <- as.matrix(read.csv("Factor_vectors.csv", header=F, stringsAsFactors = F))
abund <- read.csv("Counts_corrected.csv", header=T, stringsAsFactors = F)

target.corr <- list()
n.corr <- dim(fac.vec)[1]
n.spp <- dim(fac.vec)[2]
for(k in 1:n.corr) {
  vec<-fac.vec[k,]
  target <- matrix(0.0, n.spp, n.spp)
  for(i in 1:n.spp) {
    target[vec[i]==vec,i] <- 0.4
    }
  diag(target) <- 1.0
  target.corr[[k]] <- target
}

corr.fac <- list()
eigen.fac <- list()
boral.fac <- list()

for(k in 1:n.corr) {
  Factor.Data <- GenDataSampleMod(abund, target.corr[[k]], n.factors=k+1, max.trials=10,
        initial.multiplier=1, corr.type = "pearson", seed = 0)
  corr.fac[[k]] <- rcorr(as.matrix(Factor.Data), type="pearson")$r
  eigen.fac[[k]] <- eigen(corr.fac[[k]])$values
  boral.fac[[k]] <- boral(y = Factor.Data, family = "negative.binomial", lv.control = list(num.lv = 2), row.eff =
        "fixed", raneff.ids = matrix(rep(1:12, each=10), ncol=1))
}
eigen(rcorr(as.matrix(abund), type="pearson")$r)$values
eigen.target <- list()
for(k in 1:n.corr) eigen.target[[k]] <- eigen(target.corr[[k]])$values

corr.list <- list()
```

```
for(k in 1:n.corr) corr.list[[k]]<- corr.fac[[k]][lower.tri(corr.fac[[k]])]
corr.list

data.corr <- rcorr(as.matrix(abund) , type="pearson")$r
data.corr[lower.tri(data.corr)]

summary(boral.fac[[1]])
summary(boral.fac[[2]])
summary(boral.fac[[3]])
summary(boral.fac[[4]])
```
