# Supplementary material for: Organization of the macroinvertebrate community in a tropical annual agroecosystem into modules
Source: PLoS One. 2023 Aug 3;18(8):e0289103. doi: 10.1371/journal.pone.0289103 (PMC10399829; doi:10.1371/journal.pone.0289103)
Supplement: S1 Table — Bold are minimum AIC for each row. (PDF) [file pone.0289103.s005.pdf]

S1 Table. **AIC calculated at parameter medians[1].**

	Number of Latent Variables				
	0	1	2	3	4
Random sample effect	17320.808	16996.419	16911.295	**16879.96**	16925.904
% converging[2]	100.00	99.56	100.00	100.00	99.87
Random sample effect Nested farms	17318.087	17059.192	**16888.916**	16945.193	16904.332
% converging	100.00	100.00	100.00	100.00	100.00
Fixed sample effect	17258.028	17022.737	**16840.144**	16887.512	16878.257
% converging	99.48	99.71	99.80	99.85	99.88
Fixed sample effect Nested farms	17428.118	17074.315	**16924.764**	16943.674	16956.165
% converging	99.48	99.71	99.80	99.85	99.88

Bold are minimum AIC for each row.

[1] Selection criteria: The two latent variable model was selected based on the AIC results. We selected the fixed sample model because we were most interested in analyzing community composition and the random sample model (which can be interpreted as allowing a quadratic species response, Jamil and ter Braak 2013) did not seem essential. We selected the nested farms even though the AIC was highest because (1) the implementation of AIC in boral does not have a theoretical basis and we wanted to allow spatial variation in the colonization dynamic (species pool, colonization rates) that varied by farm and year.

[2] Gewenke convergence criterion with Holm correction of *p*-values.
